# Neuromodulatory Treatment for Dizziness and Chronic Neurological Dysfunction in an Elderly Patient Using Radio Electric Asymmetric Conveyer (REAC) Neuro Psycho Physical Optimization (NPPO) Gamma Brain Wave Optimization (BWO-G): A Case Report

**DOI:** 10.7759/cureus.77518

**Published:** 2025-01-15

**Authors:** Salvatore Rinaldi, Valeria Modestto', Arianna Rinaldi, Roseli Bittar, Jeanne Oiticica, Vania Fontani

**Affiliations:** 1 Department of Research, Department of Regenerative Medicine, Rinaldi Fontani Foundation, Florence, ITA; 2 Department of Research, Rinaldi Fontani Foundation, Florence, ITA; 3 Department of Research, Department of Regenerative Medicine, Rinaldi Fontani Institute, Florence, ITA; 4 Department of Adaptive Neuro Psycho Physio Pathology and Neuro Psycho Physical Optimization, Rinaldi Fontani Institute, Florence, ITA; 5 Department of Otolaryngology, University of São Paulo School of Medicine, São Paulo, BRA

**Keywords:** chronic neurological dysfunction, cognitive and motor recovery, dizziness, elderly patients, gamma brain wave optimization (bwo-g), neuro psycho physical optimization (nppo), noninvasive neuromodulation, radio electric asymmetric conveyer (reac)

## Abstract

An 88-year-old woman presented with a longstanding history of dizziness, tremors, and progressive mental and physical decline, significantly impairing her mobility and autonomy. Recently discharged from an ICU, the patient required extensive support for daily activities. Diagnostic evaluations, including EEG and analysis, revealed irregular frequency peaks and altered cortical activity, particularly in the frontal and prefrontal regions. The patient underwent a cycle of 18 sessions of radio electric asymmetric conveyer (REAC) Neuro Psycho Physical Optimization (NPPO) gamma brain wave optimization (BWO-G), a neuromodulatory treatment aimed at restoring neurophysiological balance. Post-treatment, the patient demonstrated marked clinical improvements, including enhanced gait stability, reduced tremors, and improved cognitive function. Electroencephalography (EEG) and standardized low-resolution brain electromagnetic tomography (sLORETA) analysis confirmed these clinical improvements, showing normalized frequency peaks and improved cortical activity patterns in Brodmann areas 6, 24, 31, 4, and 32. This case highlights the potential of REAC NPPO BWO-G in addressing chronic neurological dysfunction and improving the quality of life in elderly patients. Furthermore, the broader applicability of this treatment suggests potential benefits for managing similar conditions in aging populations, such as Parkinson's disease, age-related cognitive decline, and post-stroke rehabilitation, where bioelectrical dysregulation plays a central role.

## Introduction

Chronic neurological symptoms in elderly populations are often characterized by dizziness [1], motor impairments [2], cognitive decline [3], and emotional and psychiatric disorders [4]. These conditions significantly impact functional independence, frequently necessitating caregiver support and diminishing quality of life [5]. Current therapeutic approaches, including pharmacological and surgical interventions, are associated with limitations such as adverse effects and variable efficacy, underscoring the need for safe, repeatable, and non-invasive alternatives like the radio electric asymmetric conveyer (REAC) Neuro Psycho Physical Optimization (NPPO) gamma brain wave optimization (BWO-G) protocol.

The REAC NPPO BWO-G protocol is distinguished by its unique mechanism of action, which involves the use of weak radioelectric fields conveyed asymmetrically. Unlike transcranial magnetic stimulation (TMS) and transcranial direct current stimulation (tDCS), which rely on high-intensity electrical currents targeting specific cortical regions [6,7], REAC NPPO BWO-G selectively interacts with areas of bioelectrical dysregulation [8,9]. This targeted modulation avoids the risk of disrupting healthy neural circuits and ensures precise neurophysiological intervention. Additionally, the pre-set parameters of the REAC protocol guarantee consistency and repeatability, enhancing its reliability in clinical applications [10,11].

While many neuromodulatory techniques focus on symptomatic relief, REAC NPPO BWO-G promotes progressive restoration of cortical synchronization and connectivity by addressing underlying bioelectrical imbalances [10,11]. This feature is particularly advantageous for elderly patients, where maintaining cortical plasticity and mitigating neurophysiological decline is crucial

This report presents the diagnostic evaluation, therapeutic intervention, and clinical outcomes of an elderly patient with chronic dizziness and neurological dysfunction treated with the REAC NPPO BWO-G protocol. Particular emphasis is placed on neurophysiological improvements visualized through standardized low-resolution brain electromagnetic tomography (sLORETA) analysis [12], demonstrating the treatment’s potential in addressing chronic neurological impairments.

## Case presentation

Case presentation

The patient was an 88-year-old woman with a longstanding history of dizziness, tremors, and progressive musculoskeletal and cognitive decline. Over the past decade, her symptoms had gradually worsened, with a marked deterioration noted in the year preceding her presentation. Her condition was further exacerbated by severe mobility limitations, requiring assistance with gait and basic activities of daily living. Clinical examination revealed significant balance difficulties and visible tremors affecting both upper extremities, which interfered with fine motor tasks. Her gait was unsteady, with an increased risk of falls. Cognitive assessments indicated slowed information processing and impaired memory retention, further contributing to her growing dependency on caregivers for everyday tasks. These findings underscored the progressive and multifaceted nature of her condition, which necessitated a comprehensive and targeted therapeutic approach.

Diagnostic assessment

The diagnostic assessment in this case was focused exclusively on quantitative electroencephalography (qEEG) with power spectra analysis (PSA) [13], independent component analysis (ICA) [14], and standardized low-resolution brain electromagnetic tomography (sLORETA) analysis [12]. These evaluations were performed because the patient's relatives sought our help to address her deteriorating overall health situation. The assessments aimed to identify bioelectrical dysregulations potentially contributing to her symptoms and to provide a foundation for the subsequent neuromodulatory treatment.

The cortical dysregulations observed through qEEG PSA [13], ICA [14], and sLORETA analyses [12] were consistent with the patient's clinical presentation. Increased delta and theta activity, prominent in pre-treatment power spectra, suggested cortical inefficiency and compensatory mechanisms that are often linked to cognitive slowing, impaired attention, and difficulties in motor planning [15,16]. These findings aligned with the patient's reported dizziness, postural instability, and cognitive decline. Similarly, reduced beta and gamma activity pointed to disrupted higher-order cortical functions, affecting motor coordination, executive processing, and sensorimotor integration [17].

The ICA analysis [14] further highlighted heightened neural activity localized to the medial frontal region, encompassing Brodmann areas 6, 24, and 32 [18]. These regions are crucial for motor planning, attention control, and emotional regulation, and their dysregulation was consistent with the observed motor impairments and mood instability. Additionally, sLORETA mapping [12] identified hyperactivity in the frontal and prefrontal cortices, indicating disrupted connectivity and executive dysfunction, which correlated with the patient's cognitive and motor deficits.

By elucidating these cortical abnormalities, the diagnostic assessments provided critical insights into the neurophysiological mechanisms underlying the patient's symptoms and served as a baseline for evaluating the therapeutic impact of REAC NPPO BWO-G treatment.

Analysis of pre-treatment power spectra

**Figure 1:**
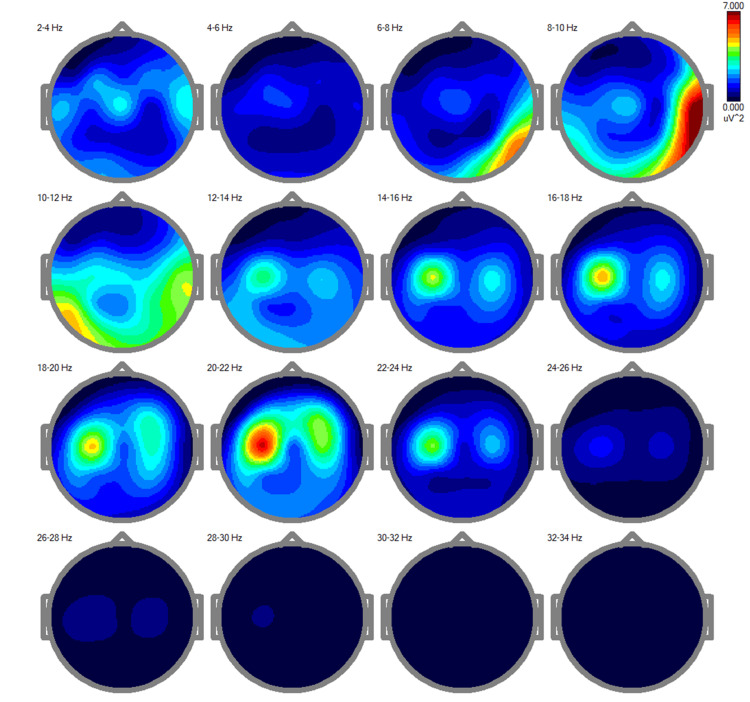
Pre-treatment EEG power spectra showing irregular cortical activity This figure illustrates maps of EEG power spectra for different frequency bands (delta, theta, alpha, beta, and gamma) during an eyes-closed session. Elevated delta and theta activity, coupled with reduced beta and gamma activity, highlight widespread cortical dysregulation associated with the patient's cognitive slowing, motor impairments, and persistent dizziness.

The pre-treatment power spectra analysis [13] revealed a clear distinct distribution of cortical activity across multiple frequency bands, ranging from 2-34 Hz (Figure 1).

Elevated activity was notably prominent in the delta (2-4 Hz) and theta (4-8 Hz) bands, which are typically associated with decreased cortical activation or compensatory mechanisms in response to inefficiencies or structural dysfunctions. This pattern is consistent with the patient's cognitive slowing and impaired motor coordination, suggesting widespread cortical dysregulation. In the alpha band (8-12 Hz), moderate activity is observed, primarily localized to posterior regions. While alpha rhythms are generally indicative of resting states and sensory processing, the patient's imbalance in alpha activity likely reflects disruptions in attentional networks, contributing to her reported dizziness and difficulty maintaining postural stability [19].

The beta activity (12-30 Hz), which plays a key role in motor control, active engagement, and executive functions, appears globally diminished [17]. This reduction is particularly pronounced in the higher beta range (18-30 Hz) and correlates with the patient's motor impairments, including tremors and unsteady gait. Gamma activity (30-34 Hz), critical for integrative processes like attention, memory, and sensorimotor coordination, is nearly absent, further highlighting impaired cortical communication and integration, likely contributing to the patient's cognitive and motor deficits.

The patterns identified in the pre-treatment power spectra closely align with the patient's clinical presentation. The elevated delta and theta activity, coupled with diminished beta and gamma rhythms, provide a neurophysiological explanation for the patient's slowed information processing, memory impairments, and motor difficulties.

The disruption in alpha rhythms, along with excessive low-frequency activity, indicates impaired sensory integration and postural maintenance, referred to as persistent dizziness [19].

Independent component analysis (pre-treatment)

**Figure 2:**
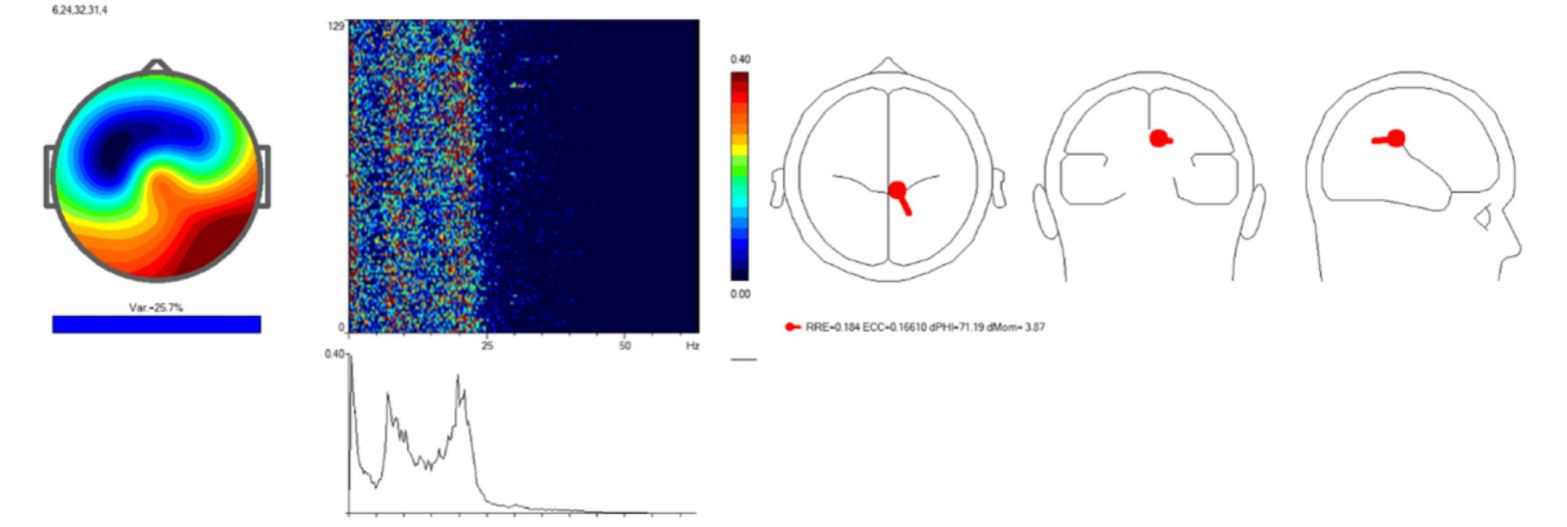
Pre-treatment Independent Component Analysis (ICA) indicating neural desynchronization The analysis reveals heightened neural activity localized to medial frontal regions, as marked by the red dot in the source localization map. This dysregulation affects key areas, including Brodmann areas 6, 24, and 32, associated with motor planning, attention control, and emotional regulation. The spectrogram and frequency spectrum demonstrate dominant low-frequency activity, correlating with the patient's motor and attentional impairments.

An independent component analysis (ICA) of the patient's EEG data was performed to identify regions of cortical dysregulation contributing to her symptoms (Figure 2).

The ICA revealed heightened neural activity, primarily localized to the medial frontal region, as indicated by the red dot in the source localization map. This region encompasses critical cortical areas, such as the anterior cingulate cortex and supplementary motor area, which are essential for motor planning, sensory integration, and attentional control.

The topographic map highlighted significant activity variance in the frontal and central regions of the scalp, corresponding to the cortical areas implicated in the patient's dizziness and motor impairments. The spectrogram and frequency spectrum revealed pronounced activity in the lower frequency ranges, particularly between 7 and 10 Hz, suggesting abnormal cortical synchronization. Such low-frequency activity is commonly linked to impaired cortical processing and functional inefficiency.

These findings provided key insights into the neurophysiological mechanisms underlying the patient's clinical presentation. The observed dysregulation of frontal and central cortical circuits was hypothesized to contribute to her dizziness, tremors, and cognitive decline. This baseline data served as a valuable reference for assessing the effectiveness of subsequent neuromodulatory treatment using the REAC NPPO BWO-G protocol.

sLORETA analysis

**Figure 3:**
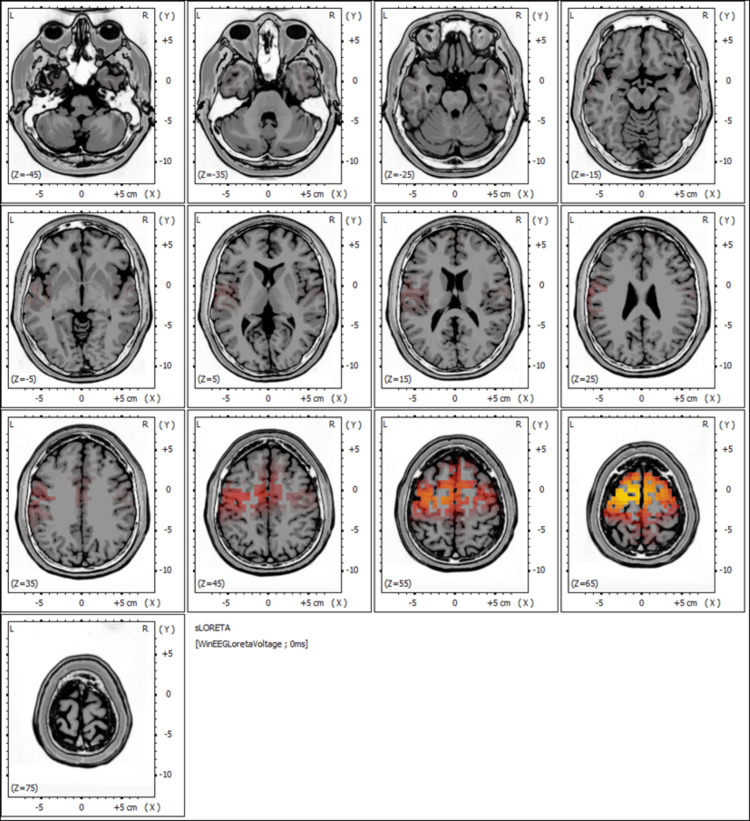
Pre-treatment sLORETA mapping highlighting regions of heightened neural activity Warmer colors (red and yellow) indicate areas of increased cortical activation, particularly in the frontal and prefrontal regions. These areas, critical for motor control, attention, and executive functions, are implicated in the patient's motor deficits, tremors, and slowed cognitive processing.

The pre-treatment EEG findings, including the sLORETA analysis shown in Figure 3, revealed regions of abnormal cortical activity, with a concentration of dysregulated neural signals in specific brain areas, as indicated by heightened activity (red to yellow intensities).

The observed patterns suggest disturbances in neural synchronization, which may potentially underlie the patient's chronic neurological symptoms, such as dizziness, tremors, and cognitive decline.

Figure 3 depicts significant activation in the frontal and prefrontal regions, which are areas typically associated with motor control, attention, and executive functions. This dysregulation aligns with the patient's clinical presentation, particularly her impaired gait, tremors, and slowed cognitive processing.

Therapeutic Intervention

The patient underwent a neuromodulatory treatment protocol using REAC NPPO BWO-G [10,11]. This protocol comprised a cycle of 18 sessions administered over six weeks, with each session lasting five minutes. The intervals between sessions were maintained at a minimum of one hour and a maximum of one week, as required, to ensure optimal therapeutic outcomes. During each session, Asymmetric Conveyor Probes (ACPs) were placed in the cervico-brachial area to facilitate the modulation of neural pathways.

The pre-set parameters of the REAC NPPO BWO-G protocol ensured uniformity in application and precision in addressing areas of cortical dysregulation [10,11]. These regions of dysfunction were identified through advanced diagnostic assessments, including EEG and sLORETA analysis [10,11], which provided detailed insights into altered bioelectrical activity. The REAC protocol's mechanism of action operates by asymmetrically modulating bioelectrical fields, promoting gradual restoration of neural synchronization and subcortical integration. This progressive reorganization specifically targeted the identified dysfunctions, contributing to the observed clinical and functional recovery.

Post-treatment power spectra analysis

**Figure 4:**
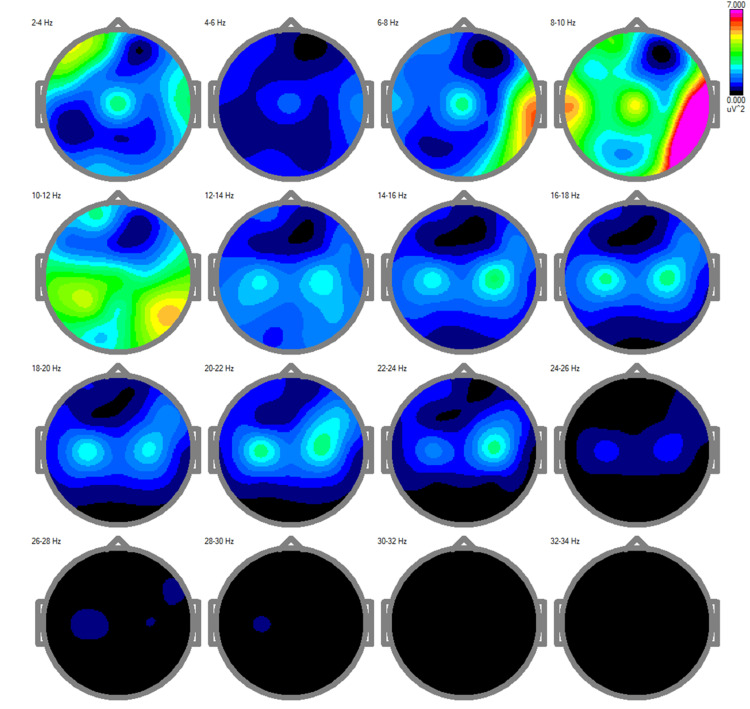
Post-treatment EEG power spectra demonstrating normalized cortical activity This figure displays the distribution of brainwave frequencies across different regions of the brain. Notable changes include: Delta (slow waves): These are prominently visible at Cz (a central scalp region in EEG recordings), indicating enhanced coordination in deep brain structures, which is typically associated with restorative processes and baseline brain function. Theta waves: Also observed at Cz, these frequencies are linked to improved memory processing and cognitive focus. Alpha waves (moderate-speed waves): Enhanced activity in the central and posterior brain areas suggests improved relaxation and attention. Alpha activity reflects a balance between relaxation and alertness, important for overall cognitive efficiency. Gamma waves (fastest waves): A slight increase in gamma activity points to improved cognitive processing, such as problem-solving and attention. These changes collectively indicate improved synchronization of brain regions, leading to better communication between them. This neurophysiological improvement aligns with the patient's clinical recovery, as normalization of these brainwave patterns is often associated with enhanced cognitive, emotional, and motor function.

The post-treatment power spectra analysis revealed significant and clinically relevant changes in cortical activity across multiple frequency bands, reflecting improvements in neural dynamics and synchronization (Figure 4).

Delta activity (2-4 Hz), characterized by the emergence of this power with a more localized distribution pattern, suggests a reduction in cortical inefficiency and compensatory low-frequency overactivation. This pattern correlates with improvements in the patient's cognitive and motor processing, as well as overall cortical function. Theta activity (4-8 Hz), while still present, exhibited a more constrained spatial pattern, indicating enhanced cortical efficiency and reduced dependency on low-frequency oscillations often associated with impaired processes.

The alpha band (8-12 Hz) displayed increased and more symmetrical activity, particularly in posterior regions, which are critical for sensory integration and attentional regulation [19]. This normalization of alpha rhythms aligns with the patient's reported improvements in dizziness and postural stability, suggesting enhanced coordination between sensory input and motor output systems [19].

Beta activity (12-30 Hz) showed a marked increase, particularly in the mid and high beta ranges (18-30 Hz). This enhancement is strongly associated with the restoration of motor planning, execution, and executive functions [17]. The increased beta activity correlates with the observed reductions in tremors and the improvement in gait stability. Gamma activity (30-34 Hz), while minimal, exhibited subtle increases post-treatment. The observed improvement in gamma activity suggests a partial restoration of higher-order integrative processes, consistent with the patient's enhanced cognitive clarity and functional independence.

These normalization patterns observed in the qEEG findings highlight the progressive restoration of bioelectrical activity within the patient's cortical networks. The changes in delta, theta, beta, and gamma activities collectively suggest a rebalancing of excitatory and inhibitory mechanisms, leading to improved neurophysiological efficiency. This neural reorganization directly correlated with the functional recovery observed clinically, including stabilized gait, reduced tremors, and enhanced cognitive performance.

Clinical correlation and significance

The post-treatment power spectra analysis demonstrated significant improvements in the patient's neurophysiological state, correlating with her clinical recovery. The marked reduction in delta and theta activity reflects diminished cortical inefficiency and reduced reliance on compensatory mechanisms, aligning with enhanced cognitive processing and motor coordination. The normalization of alpha rhythms, particularly in posterior regions, indicates improved sensory integration and attentional focus, which are essential for maintaining postural stability. The substantial increase in beta activity underscores restored motor planning and executive functions, as evidenced by reductions in tremors and enhanced gait stability. Although gamma activity exhibited only modest increases, this improvement points to the gradual restoration of higher-order cognitive processes and sensorimotor integration, further contributing to the patient's regained independence.

The comparative analysis of pre- and post-treatment findings underscores the comprehensive reorganization of cortical activity achieved through REAC NPPO BWO-G. Pre-treatment evaluations highlighted a dominance of low-frequency oscillations (delta and theta bands) and a suppression of high-frequency activity (beta and gamma bands), indicative of widespread cortical dysfunction and connectivity disruptions. Post-treatment, these imbalances were significantly mitigated, with normalized alpha rhythms supporting sensory-motor integration and enhanced beta activity correlating with improved executive and motor functions. The partial recovery of gamma activity suggests a progressive realignment of higher-order integrative processes.

Collectively, these changes highlight the ability of REAC NPPO BWO-G to address systemic bioelectrical dysregulation, fostering long-term neurophysiological recovery and functional reorganization. This capability is particularly relevant for elderly populations, where cortical plasticity and resilience are often diminished. These findings not only validate the observed clinical outcomes but also emphasize the treatment's broader applicability for conditions characterized by chronic neurophysiological decline, such as Parkinson's disease, age-related cognitive dysfunction, and post-stroke recovery.

Post-treatment ICA analysis

**Figure 5:**
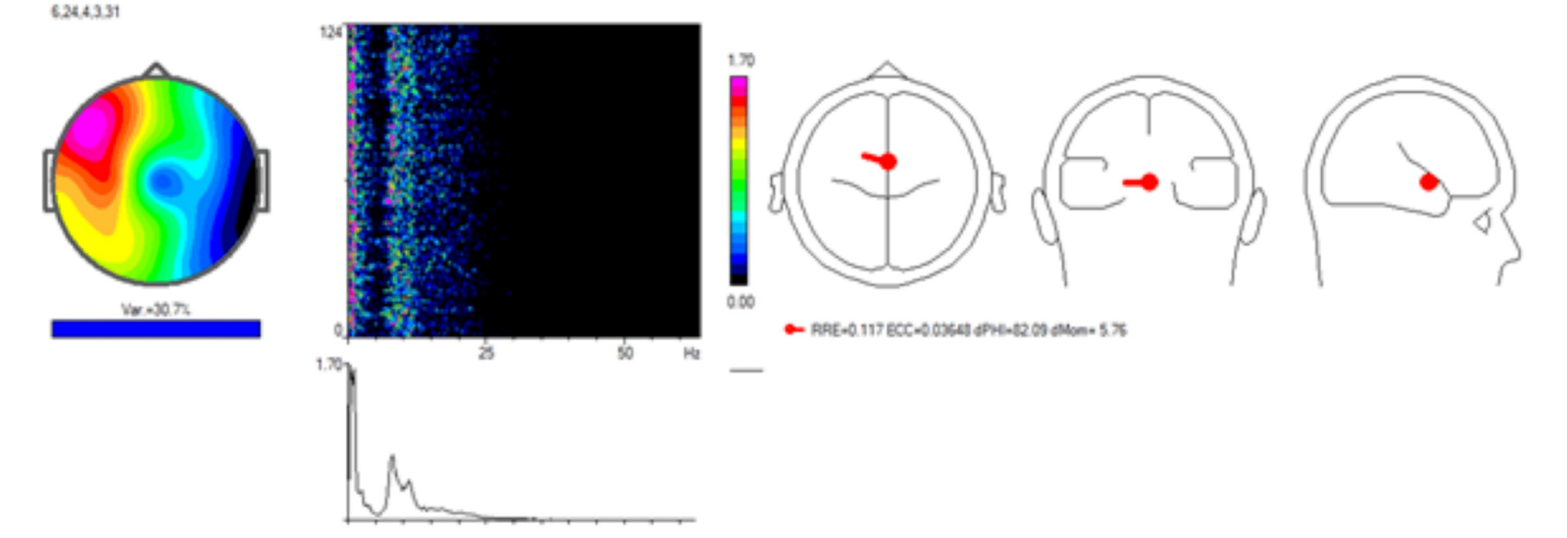
Post-treatment independent component analysis (ICA) illustrating improved neural synchronization The post-treatment ICA highlights more centralized activity with reduced variance in frontal regions, as represented by a shift in frequency peaks and decreased dominance of low-frequency activity. Relevant Brodmann areas (6, 24, 4, 3 and 31) show improved functionality, correlating with enhanced motor planning, emotional regulation, and sensorimotor integration.

The ICA performed after the treatment revealed significant changes in neural activity, reflecting improvements in cortical synchronization (Figure 5).

Source localization identified key cortical areas involved, including Brodmann areas 6, 24, 4, 3, and 31. These regions are associated with motor planning (areas 6 and 4), emotional regulation and attention (areas 24 and 31), somatosensory integration (area 3), and higher-order cognitive functions (area 31).

The topographic map displayed a more centralized and confined distribution of activity compared to the pre-treatment findings. The intensity of variance, as represented by warmer colors, was reduced, reflecting improved neural efficiency and a decrease in hyperactivity.

The frequency peaks observed post-treatment were 1.46 Hz, 7.08 Hz, 7.57 Hz, 10.99 Hz, and 23.19 Hz. Compared to the pre-treatment findings, the shift in peak frequencies suggests a normalization of neural oscillatory patterns, with a reduced prominence of low-frequency activity and a more balanced distribution across multiple frequency bands. This indicates restored cortical function and decreased compensatory overactivation.

The spectrogram demonstrated a reduction in high-power activity within the lower frequency range (below 10 Hz), which was previously associated with cortical inefficiency and dysregulation. The power spectrum revealed a more even distribution, consistent with a normalization of neural activity.

Post-treatment sLORETA analysis

**Figure 6:**
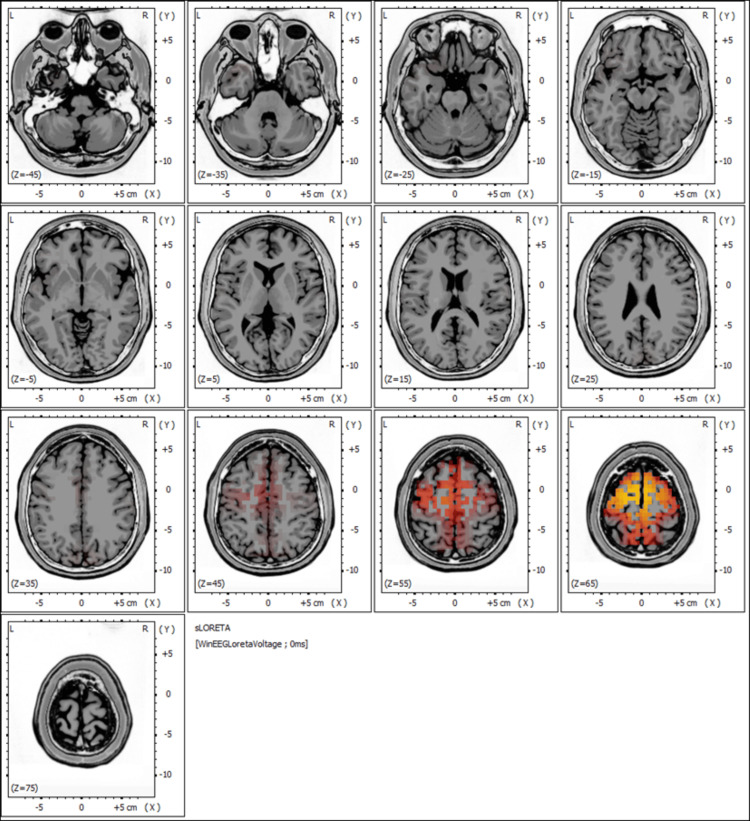
Post-treatment standardized low-resolution brain electromagnetic tomography (sLORETA) mapping showing enhanced brain activity and connectivity This post-treatment sLORETA map depicts a reduction in hyperactivity in the frontal and prefrontal regions, with fewer red and yellow areas. The normalized activity patterns reflect improved cortical synchronization, contributing to the patient’s restored motor functions, reduced tremors, and cognitive clarity.

The sLORETA analysis performed after the REAC NPPO BWO-G treatment revealed significant normalization of cortical activity patterns compared to pre-treatment findings (Figure 6).

Pre-treatment sLORETA mapping highlighted heightened activity, particularly in the frontal and prefrontal regions, which are critical for motor control, executive functions, and attention regulation. This hyperactivation was consistent with the patient's clinical presentation of dizziness, tremors, impaired gait, and slowed cognitive processing.

Post-treatment mapping demonstrated marked reductions in hyperactivity within these regions. The frontal cortices, responsible for motor planning and execution, exhibited a more balanced activity pattern, correlating with the patient's improved gait stability and reduced tremors. Similarly, the prefrontal regions, which govern executive and cognitive functions, showed a normalization of activity, reflecting enhanced memory retention, cognitive clarity, and reduced reliance on compensatory mechanisms.

The improved synchronization of cortical activity was evidenced by a decrease in the dominance of low-frequency oscillations and a more even distribution of neural activity across higher-frequency bands. This reorganization of cortical dynamics indicated restored neural efficiency and connectivity, essential for sensorimotor integration and cognitive processing.

These findings provide compelling evidence of the REAC NPPO BWO-G protocol's capacity to address bioelectrical dysregulation, promoting cortical reorganization and neurophysiological recovery. The observed improvements in cortical synchronization are directly linked to the patient's functional recovery, including enhanced motor control, cognitive function, and overall independence.

Clinical implications of sLORETA and ICA analyses

The post-treatment ICA and sLORETA analyses demonstrated significant improvements in cortical activity and synchronization, correlating with the patient's clinical recovery.

The ICA analysis revealed more centralized and confined neural activity compared to pre-treatment findings. Variance in frontal regions, particularly Brodmann areas 6, 24, 31, 4, and 32, was markedly reduced. These areas, critical for motor planning, emotional regulation, and sensorimotor integration, exhibited enhanced functionality. The frequency spectrum showed a shift towards higher frequencies, indicating reduced dominance of low-frequency oscillations associated with cortical inefficiency. This normalization of neural activity aligns with the patient's improved motor coordination and emotional stability.

The sLORETA analysis further confirmed these findings, showing decreased hyperactivity in the frontal and prefrontal cortices. Reduced activity in these regions was associated with better executive functions, improved memory retention, and enhanced gait stability. The post-treatment mapping highlighted a more even distribution of neural activity across frequency bands, particularly beta and gamma. These changes reflect improved cortical synchronization and connectivity, essential for cognitive processing and motor execution.

Together, the ICA and sLORETA analyses underscore the ability of REAC NPPO BWO-G to address bioelectrical dysregulation and promote neurophysiological recovery. The observed improvements in cortical synchronization and efficiency were directly linked to the patient's functional recovery, including enhanced independence and quality of life.

Follow-up and outcomes

Throughout the course of treatment, the patient exhibited steady and substantial improvements. Quantitative assessments revealed that gait stability improved by approximately 65%, as measured by the reduction in episodes of imbalance during a 10-meter walking test. Tremors, which previously interfered with basic tasks, were reduced by approximately 70%, enabling her to regain significant manual dexterity. These improvements were assessed by caregiver-reported metrics, including increased ease in tasks such as holding utensils and buttoning clothing. Cognitively, family members and caregivers reported a marked increase in her memory retention and conversational engagement, estimated at a 50% improvement. This was supported by improved caregiver-reported observations of her ability to recall recent events and actively participate in discussions.

From the caregivers' perspective, these changes significantly reduced the burden of daily assistance. The patient's independence in performing self-care tasks, such as dressing and eating, showed a 75% overall improvement compared to their complete dependence prior to treatment, as observed through the Katz Index of Independence in Activities of Daily Living (ADL) [20]. This progress was also reflected in a healthy increase in body weight, indicating improved nutritional status.

Caregivers also noted a dramatic improvement in her mood and responsiveness, describing her as "more lively and engaged" in daily activities. These observations were supported by family feedback, which highlighted the patient's increased involvement in social interactions and greater enthusiasm for participating in routine activities. The follow-up EEG corroborated these findings, revealing a marked normalization of frequency peaks and enhanced cortical activity, suggesting successful neuromodulation (Figures 4-6).

## Discussion

This case report highlights the significant clinical and neurophysiological improvements observed in an elderly patient with chronic neurological dysfunctions following REAC NPPO BWO-G treatment. The findings demonstrate the clinical efficacy of this innovative neuromodulation protocol and its broader potential for addressing cortical dysregulation and fostering neurophysiological recovery in aging populations.

Unique mechanism of action

Unlike traditional neuromodulatory approaches, such as TMS or transcranial direct current stimulation (tDCS) [6,7], REAC NPPO BWO-G employs weak radioelectric fields to asymmetrically interact with bioelectrical imbalances in cortical and subcortical regions [10,11]. This mechanism avoids introducing high-intensity external currents that may disrupt healthy neural circuits, making it particularly suitable for elderly patients. By facilitating endogenous recovery processes, REAC NPPO BWO-G promotes neural synchronization and connectivity, addressing the root causes of cortical dysregulation rather than merely alleviating symptoms.

Sustained and amplified cortical plasticity with repeated treatment cycles

One of the most promising aspects of REAC NPPO BWO-G is its capacity to enhance cortical plasticity and neurophysiological recovery over repeated treatment cycles. In elderly populations, where plasticity is often diminished due to age-related neurodegeneration, the iterative nature of the treatment allows for progressive improvements in cognitive, motor, and emotional functions [10,11]. Administering treatment cycles approximately every six months provides an opportunity to sustain the benefits achieved in initial sessions and potentially amplify long-term neurophysiological adaptations.

Repeated cycles may further promote the reorganization of neural networks, enhancing the functional integration of sensory, motor, and cognitive processes. This adaptability is critical for maintaining quality of life and functional independence in aging individuals. However, the cumulative effects of repeated cycles require systematic investigation to establish optimal intervals and to understand potential diminishing returns.

Comparative advantages and future directions

Compared to other neuromodulatory treatments [6,7], REAC NPPO BWO-G stands out for its precision, safety, and ability to address systemic bioelectrical dysregulation rather than localized dysfunction. Its operator-independent, pre-set parameters ensure consistency, making it a reliable option for clinical implementation.

Future studies should focus on evaluating the long-term effects of repeated treatment cycles, including their impact on cortical plasticity, functional recovery, and quality of life. Expanding the evidence base through larger cohorts and diverse patient populations will further validate the versatility of REAC NPPO BWO-G. Additionally, exploring the treatment's integration with conventional therapies could provide synergistic benefits for managing complex neurological disorders.

## Conclusions

This case report demonstrates the potential of REAC NPPO BWO-G as a transformative neuromodulatory treatment for chronic neurological dysfunctions in elderly patients. By addressing bioelectrical dysregulation and restoring cortical balance, this non-invasive approach offers remarkable clinical benefits, including enhanced motor function, improved cognitive clarity, and greater independence.

While the observed outcomes are promising, further research is essential to validate these findings and explore their applicability across diverse patient populations. Expanding the evidence base through larger studies will help establish REAC NPPO BWO-G as a reliable option for addressing chronic neurophysiological dysfunctions. Additionally, investigating the cumulative effects of repeated treatment cycles could provide insights into maximizing long-term benefits, particularly for conditions characterized by progressive cortical decline, such as Parkinson's disease, age-related cognitive impairments, and post-stroke recovery.

The integration of REAC NPPO BWO-G into clinical practice highlights its promise as an innovative solution for managing neurological challenges associated with aging. By targeting underlying bioelectrical imbalances, this treatment provides a feasible and effective alternative to more invasive or symptom-focused neuromodulatory approaches. The substantial recovery observed in this patient underscores the importance of advancing research and refining neuromodulatory techniques for broader clinical use.

## References

[1] Fancello V, Hatzopoulos S, Santopietro G (2023). Vertigo in the elderly: a systematic literature review. J Clin Med.

[2] Roca F, Lang PO, Chassagne P (2019). Chronic neurological disorders and related comorbidities: Role of age-associated physiological changes. Handb Clin Neurol.

[3] Grande G, Vetrano DL, Kalpouzos G (2023). Brain changes and fast cognitive and motor decline in older adults. J Gerontol A Biol Sci Med Sci.

[4] Ismail Z, Gatchel J, Bateman DR (2018). Affective and emotional dysregulation as pre-dementia risk markers: exploring the mild behavioral impairment symptoms of depression, anxiety, irritability, and euphoria. Int Psychogeriatr.

[5] Reis Júnior WM, Ferreira LN, Molina-Bastos CG, Bispo Júnior JP, Reis HF, Goulart BN (2024). Prevalence of functional dependence and chronic diseases in the community-dwelling Brazilian older adults: an analysis by dependence severity and multimorbidity pattern. BMC Public Health.

[6] Siebner HR, Funke K, Aberra AS (2022). Transcranial magnetic stimulation of the brain: What is stimulated? - A consensus and critical position paper. Clin Neurophysiol.

[7] Vergallito A, Varoli E, Pisoni A (2023). State-dependent effectiveness of cathodal transcranial direct current stimulation on cortical excitability. Neuroimage.

[8] Rinaldi S, Mura M, Castagna A, Fontani V (2014). Long-lasting changes in brain activation induced by a single REAC technology pulse in Wi-Fi bands. Randomized double-blind fMRI qualitative study. Sci Rep.

[9] André Nogueira JA, Souza Bulle Oliveira A, Pereira Motta M (2024). Neurobiological modulation with REAC technology: enhancing pain, depression, anxiety, stress, and quality of life in post-polio syndrome subjects. Sci Rep.

[10] Modesto' V, Rinaldi A, Fontani V, Rinaldi S (2024). Efficacy of Radio Electric Asymmetric conveyer neuro psycho physical optimization - brain wave optimization-gamma (REAC NPPO BWO-G) treatment in neurovegetative dysfunction: a case report of enhanced cognitive processing and stress resilience. Cureus.

[11] Modesto' V, Rinaldi A, Fontani V, Rinaldi S (2024). Non-invasive gamma brain wave optimization (BWO-G) for cognitive and emotional recovery in an adolescent: a case study on Radio Electric Asymmetric conveyer (REAC) neuro psycho physical optimization (NPPO) BWO-G treatment. Cureus.

[12] Moon JU, Lee JY, Kim KY, Eom TH, Kim YH, Lee IG (2022). Comparative analysis of background EEG activity in juvenile myoclonic epilepsy during valproic acid treatment: a standardized, low-resolution, brain electromagnetic tomography (sLORETA) study. BMC Neurol.

[13] Pani SM, Saba L, Fraschini M (2022). Clinical applications of EEG power spectra aperiodic component analysis: a mini-review. Clin Neurophysiol.

[14] Habib MA, Ibrahim F, Mohktar MS, Kamaruzzaman SB, Lim KS (2020). Recursive independent component analysis (ICA)-decomposition of ictal EEG to select the best ictal component for EEG source imaging. Clin Neurophysiol.

[15] Tan E, Troller-Renfree SV, Morales S, Buzzell GA, McSweeney M, Antúnez M, Fox NA (2024). Theta activity and cognitive functioning: Integrating evidence from resting-state and task-related developmental electroencephalography (EEG) research. Dev Cogn Neurosci.

[16] Kaushik P, Moye A, Vugt MV, Roy PP (2022). Decoding the cognitive states of attention and distraction in a real-life setting using EEG. Sci Rep.

[17] Barone J, Rossiter HE (2021). Understanding the role of sensorimotor beta oscillations. Front Syst Neurosci.

[18] Zilles K (2018). Brodmann: a pioneer of human brain mapping-his impact on concepts of cortical organization. Brain.

[19] Edwards AE, Guven O, Furman MD, Arshad Q, Bronstein AM (2018). Electroencephalographic correlates of continuous postural tasks of increasing difficulty. Neuroscience.

[20] Lino VT, Pereira SR, Camacho LA, Ribeiro Filho ST, Buksman S (2008). Cross-cultural adaptation of the Independence in Activities of Daily Living Index (Katz Index) (Article In Portugese). Cad Saude Publica.

